# The Potential of Bovine Colostrum-Derived Exosomes to Repair Aged and Damaged Skin Cells

**DOI:** 10.3390/pharmaceutics14020307

**Published:** 2022-01-27

**Authors:** Geonhee Han, Hyosuk Kim, Da Eun Kim, Yeonjoo Ahn, Joongsoo Kim, Ye Ji Jang, Kwangmeyung Kim, Yoosoo Yang, Sun Hwa Kim

**Affiliations:** 1 KU-KIST Graduate School of Converging Science and Technology, Korea University, 145 Anam-ro, Seoul 02841, Korea; geonhee@kist.re.kr (G.H.); kim@kist.re.kr (K.K.); 2 Center for Theragnosis, Biomedical Research Institute, Korea Institute of Science and Technology, Hwarangno 14-gil 5, Seoul 02792, Korea; hyoseog7@kist.re.kr (H.K.); g19501@kist.re.kr (D.E.K.); 3 Division of Bio-Medical Science and Technology, KIST School, Korea University of Science and Technology, Seoul 02792, Korea; 4 Dayone Clinic, Seoul 06612, Korea; yeonjoo.an@gmail.com (Y.A.); fmdr.without.border@gmail.com (J.K.); 5 HB Advisors, Seoul 04420, Korea; yjjang@tdinv.co.kr

**Keywords:** bovine colostrum, exosome, keratinocyte, melanocyte, fibroblast, skin repair

## Abstract

In this study, we examined the potentially beneficial effects of bovine colostrum-derived exosomes on UV-induced aging and damage in three major resident skin cells including keratinocytes, melanocytes, and fibroblasts. The treatment with colostrum exosomes prevented the UV-induced generation of intracellular reactive oxygen species in epidermal keratinocytes. In UV-stimulated melanocytes, colostrum exosomes could also significantly reduce the production of the protective skin-darkening pigment melanin, which may help to reduce the risk of excessive melanin formation causing skin hyperpigmentation disorders. In the human dermal fibroblasts treated with colostrum exosomes, the expression of matrix metalloproteinases was suppressed, whereas increased cell proliferation was accompanied by enhanced production of collagen, a major extracellular matrix component of skin. Taken together, our findings indicate that bovine colostrum-derived exosomes having excellent structural and functional stability offer great potential as natural therapeutic agents to repair UV-irradiated skin aging and damage.

## 1. Introduction

Skin aging is caused by both intrinsic (genetics) and extrinsic (mental stress, gut microbiome, air pollution, food, and UV exposure) factors [1,2,3]. In particular, UV light is the most common environmental stress to aging and damaging skin cells, and in severe cases it acts as a mutagenic substance leading to cutaneous cancer [4]. UV can react with water molecules in the skin to produce reactive oxygen species (ROS) that cause cell apoptosis and gene mutations through oxidative damage to lipids, proteins, and DNA [5,6,7]. Up to date, many efforts have been made to apply stem cells to the development of novel therapeutic strategies to achieve skin regeneration and repair [8,9]. However, there are various challenges such as low engraftment rate of transplanted cells, and uncontrollable proliferation and differentiation of cells, etc. More recently, exosomes extracted from various stem cells have attracted attention as potential alternatives to stem cell therapy for skin wound healing due to their ability to provide therapeutic benefits without the limitations and risks of stem cells [10,11,12].

Exosomes are a type of membrane-bound extracellular vesicle (EV) secreted by cells for intercellular communication [13]. They are nano-sized particles about 30 to 150 nm formed into a spherical lipid bilayer that carries the various lipids, proteins, and nucleic acids produced by parent cells. Therefore, exosomes are small enough to allow filtration to ensure product sterility and safety, and stable enough for long-term frozen storage. In addition, current animal studies report the notable safety of the vesicles by showing no adverse reaction with a large overdosage (200–250 μg) [14,15,16,17]. Due to these characteristics, stem cell-derived exosomes are widely used as materials for regenerative medicine [18,19]. Furthermore, exosomes have the ability to penetrate the stratum corneum during skin treatment and deliver the contents of the exosomes to the dermis [20]. However, limitation for scaling up cell culture and low separation yield of exosomes impede the use of cultured animal cells, including stem cells as the biological sources of exosomes. Unlike animal cell lines, natural biological fluids such as plasma and breast milk are well known as richer sources of exosomes [21,22].

Bovine milk is an easily accessible mass source of exosomes, which can be isolated in super-high yields using methods such as ultra-centrifugation (UC) and isoelectric precipitation (IP) [22]. Milk as a rich nutrient has been used in skincare and healthcare for centuries. This solution is absorbed through the gastrointestinal tract (GI) and is involved in the growth, development, nutrition, and differentiation of primary keratinocytes in newborns [23,24]. Recent studies have found that bovine milk is able to promote healing of full thickness wounds with increased fibroblasts and collagen fibers [25,26,27]. In particular, colostrum is typically more enriched in immune and bioactive factors than mature milk made during lactation. The efficacy of colostrum is widely known but the correlation between bovine colostrum-derived exosomes (Col M-exos) and skin was unclear. In this study, we investigated the effects and properties of milk exosomes, especially Col M-exos, on skin cells. Our findings demonstrated that Col M-exos, having excellent structural and functional stability, could increase collagen production while reducing ROS and melanin production in the various cell types present in the skin, suggesting the potential applicability of milk exosomes in advanced cell-free skin regeneration therapy.

## 2. Materials and Methods

### 2.1. Exosome Isolation

The colostrum used in the experiment was milk provided from the Cheongsol farm (Naju, Korea) and was collected from cows within 7 days of them giving birth to calves. For the mature milk, pasteurized milk was used (Sangha farm, Gwangju, Korea). The milk impurities were filtered with a cell strainer and centrifuged at 5000× *g* for 30 min and 12,000× *g* for 1 h continuously. The upper layer of fat was removed and only the supernatant was taken. Thereafter, the supernatant was ultracentrifuged at 35,000× *g* and 70,000× *g* for 1 and 3 h, respectively, using an ultracentrifuge (Optima XE-100, Beckman Coulter, Brea, CA, USA). Only the middle layer solution was extracted, excluding the fat layer and milk protein pellets. The solution was passed sequentially through filters of 0.8, 0.45, and 0.2 μm pore sizes to remove any remaining whey proteins and bacteria. The passed solution was ultracentrifuged for 1 h at 100,000× *g* to make milk exosome pellets. The formed pellet was washed several times with phosphate-buffered saline (PBS) and then dissolved in PBS.

Exosomes extracted from the cell medium were isolated using the ultracentrifugation method as previously described [28]. The cells were incubated for 48 h in serum-free conditions. Next, to remove dead cells, the assembled medium was centrifuged at 300× *g* for 10 min and then centrifuged at 2000× *g* for 10 min. Sequentially, the medium was centrifuged at 10,000× *g* for 30 min to remove cell debris and the supernatant was harvested. This supernatant was ultracentrifuged at 150,000× *g* for 3 h to collect the exosome pellet. This pellet was washed several times with PBS and then dissolved in PBS. All processes of extracting exosomes were performed at 4 °C.

### 2.2. Exosome Characterization

Measurement of exosome size was performed using DLS (Zetasizer Nano ZS, Malvern Instruments, Malvern, UK). For TEM (Tecnai F20, FEI Company, Hillsboro, OR, USA) analysis of exosomes, we used a 2% paraformaldehyde solution overnight. Exosome solution was centrifuged for 30 min at 150,000× *g* and then suspended in absolute ethanol. A total of 10 µg of exosome suspension was relocated onto Formvar-carbon coated electron microscopy grids. To contrast, the grid was treated with uranium acetate solution for 30 s and then examined using TEM.

### 2.3. Cell Culture and UV-C Irradiation

For proliferation assay and ROS estimation assay, Human keratinocyte cells, HaCaT, (ATCC CRL-2404, Manassas, VA, USA) were used. Melanoma, B16F10, (ATCC CRL-6475, Manassas, VA, USA) was used to perform melanin assay. In addition, human dermal fibroblast cells, HDFs, (ATCC PCS-201-012, Manassas, VA, USA) were used to conduct the confirmation of cytotoxicity and cellular uptake of exosomes, proliferation assay, and Immunofluorescence. HaCaTs and HDFs were cultured in DMEM medium (CM002-050, GenDEPOT, Katy, TX, USA) added with 10% FBS (F0600-050, GenDEPOT, Katy, TX, USA), 1% Antibiotic-Antimycotic (CA002-010, GenDEPOT, Katy, TX, USA) at 37 °C 5% CO_2_. B16F10s were cultured in RPMI 1640 medium (CM059-050, GenDEPOT, Katy, TX, USA) added with 10% FBS, 1% antibiotic-antimycotic at 37 °C and 5% CO_2_. The cells to be irradiated with UV-C were washed with PBS and then irradiated with UV-C for the necessary time using a UV-C lamp (G40T10, Sankyo-Denki, Kanagawa, Yokohama, Japan). Immediately after UV-C irradiation, the cells were cultured in their respective media.

### 2.4. Cell Proliferation Assay

HaCaTs and HDFs were plated on 96 well culture dishes at a density of 5 × 10^3^ cells/well in the fresh culture medium. Cells were incubated for 6 h to allow them to attach to the bottom. Subsequently, the cells were irradiated with 4.8 mJ/cm^2^ of UV-C (if necessary) and cells were treated with exosomes at a concentration of 0.1 mg/mL for 24 h, then 10 μL of Cell Counting Kit-8 solution (CCK-8) (CK04, Dojindo Laboratories, Kumamoto, Japan) was added to the well, and the cells were incubated for an additional 25 min.

### 2.5. Exosome Labeling and Cellular Uptake

Col M-exo was labeled Cyanine5.5-N-hydroxysuccinimide (Cy5.5-NHS) ester (#67020, Lumiprobe, Hunt Valley, MD, USA) regarding the manufacturer’s protocol. The exosomes and Cy5.5-NHS-ester were mixed and incubated overnight. To remove the unattached Cy5.5-NHS-ester, the mixture was centrifuged at 20 psi using Airfuge Air-Driven Ultracentrifuge (340400, Beckman Coulter, Brea, CA, USA) and the supernatant was discarded. This washing step was repeated several times. HDFs were seeded in a 35 mm confocal dish at a density of 2 × 10^5^ cells per dish. The cells were treated with labeled exosomes (0.05, 0.1 mg/mL) and incubated for 1, 4, 16, and 24 h. Then the cells were observed with a confocal microscope (TCS SP8, Leica, Wetzlar, Germany). The fluorescence intensities were evaluated using an image analyzer.

### 2.6. Apoptosis Analysis

Apoptosis analysis of HDFs was conducted by flow cytometry (Guava easyCyte Flow Cytometers, Millipore, Burlington, VT, USA) using an Annexin V-FITC Apoptosis Detection Kit (APOAF-50TST, Sigma-Aldrich, St. Louis, MI, USA). The trypsinized cells were washed with PBS and resuspended in 1× Binding Buffer at a concentration of 10^6^ cells/mL. A total of 5 μL of Annexin V and 5 μL of PI Staining Solution were added to the 100 μL of the cell suspension solution. Then, the cells were incubated for 10 min at room temperature while blocking the light. Next, the cells were centrifuged at 500× *g* for 5 min. The supernatant was discarded and then the cells were re-suspended in 200 μL of 1× Binding Buffer. The stained cells were analyzed by flow cytometry.

### 2.7. Western Blotting

Each type of exosomes and cells were solubilized by incubating on ice with RIPA buffer (89900, Thermo Fisher SCIENTIFIC, Waltham, MA, USA) including 1% protease inhibitors. To remove cell debris, the lysates were centrifuged at 12,000 rpm for 20 min. After the centrifugation step, supernatants were quantitated by bicinchoninic acid protein assay. The lysates were electrophoresed with 10% sodium dodecyl sulfate-polyacrylamide gel. Then, proteins were transferred to nitrocellulose membrane and the membrane was blocked with 5% skim milk solution for 30 min at room temperature. After blocking, the membrane was incubated with primary antibody overnight at 4 °C. The membrane was washed several times for 15 min with TBS-T buffer (Tris-buffered saline with 0.1% Tween 20). After washing, the membrane was incubated in 5% skim milk solution with second antibody-tagged horseradish peroxidase for 1 h at room temperature. The labeled proteins were visualized using a luminescent image analyzer (LAS-3000, FujiFilm, Tokyo, Japan). Information of the antibodies used in this study are as follows: Alix (ab117600, 1:1000, Abcam, Cambridge, UK), Tsg101(ab83, 1:1000, Abcam, Cambridge, UK), MFG-E8 (AF2805, 1:1000, Minneapolis, R&D Systems, Minneapolis, MN, USA), GSH (ab19534, 1:1000, Abcam, Cambridge, UK), β-actin (ab8227, 1:1000, Abcam, Cambridge, UK), and MMP2 (#4022, 1:200, Danvers, Cell Signaling Technology, Danvers, Massachusetts, USA).

### 2.8. ROS Estimation

Oxidative stress was measured by detecting intracellular ROS of HaCaTs. ROS was estimated by 2′,7′-dichlorofluorescein diacetate (D6883, Merck, Darmstadt, Germany), which reacts with intracellular oxidative ROS and is converted to dichlorofluorescein (DCF). The cells were seeded at a density of 2.5 × 10^5^ cells/dish and treated with HaCaT-, mature milk- or colostrum-derived exosomes at a concentration of 0.05 mg/mL overnight. As a positive control, the cells were pretreated with 100 μM hydrogen peroxide for 30 min. The cells were irradiated with 0.81 mJ/cm^2^ of UV-C (if necessary), and after 6 h, the cells were incubated with DCF-DA at a concentration of 50 μM for 30 min. The cells were washed once with PBS and observed with a confocal microscope (TCS SP8, Leica, Wetzlar, Germany).

### 2.9. Measurement of Melanin Content

B16F10s were seeded at a density of 1 × 10^6^ cell/dish. The cells were treated with B16F10 exosomes, mature milk exosome or colostrum exosome at a concentration of 0, 0.002, 0.005, 0.01, or 0.02 mg/mL for 24 h, and co-treated with 100 nM α-MSH (M4135, Merck, Darmstadt, Germany) for 24 h. After 24 h, the cells were irradiated with 0.41 mJ/cm^2^ of UV-C and treated with exosomes and α-MSH at the same concentration as the previous day. Then, after 24 h, the same number of cells was counted and harvested. The cells were washed with PBS and melanin was eluted with a solution of 1 N NaOH containing 10% DMSO for 1 h at 80 °C. Each sample was transferred to a 96-well microplate and ODs were detected at 490 nm.

### 2.10. Immunofluorescence

HDFs were seeded at a density of 2 × 10^5^ cells/dish. The cells were treated with each exosome (0.1 mg/mL) and incubated for 24 h. Then, cells were rinsed with PBS and fixed with 4% paraformaldehyde for 10 min. After fixation, cells were washed three times with ice-cold PBS and permeabilized with 0.25% Triton X-100 (PBST) for 5 min. Blocking with PBST solution containing 1% Bovine Serum Albumin (BSA) was performed for 30 min, and primary antibodies were added in blocking solution overnight at 4 °C. The cells were washed three times with PBST and then the secondary antibodies were incubated in the dark for 1 h at room temperature. After three PBST washes, DAPI staining was performed. The images were taken using a confocal microscope (TCS SP8, Leica, Wetzlar, Germany). The antibodies used in this experiment are as follows: type 1 collagen (Abcam, Cambridge, UK, ab260043, 1:200), Alexa Fluor^®^ 488 (Abcam, ab150077, 1:500).

### 2.11. Freeze Drying

Exosomes dissolved in PBS were freeze-dried overnight using a freeze dryer (FD8508, Ilshinbiobase, Gyeonggi-do, Yangju, Korea) and stored at room temperature for 2 days. Before using exosome, exosomes were dissolved in deionized water overnight.

### 2.12. Statistical Analysis

All data were demonstrated as mean ± SD in at least three independent experiments, and *p*-values less than 0.05 were recognized as statistically significant differences in the data. Data were analyzed using Student *t*-test or one-way analysis of variance, and multiple comparisons were calculated using a Tukey–Kramer post hoc test.

## 3. Results and Discussion

### 3.1. Preparation and Characterization of Milk Exosomes

**Figure 1:**
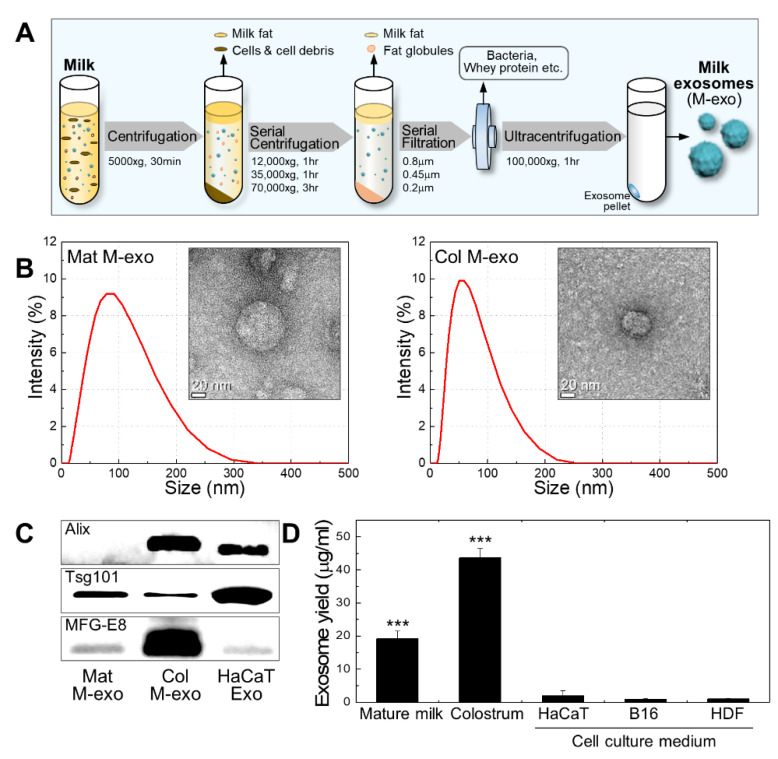
Extraction method and characterization of milk exosomes. (**A**) Schematic diagram of a method for extracting exosomes from milk. (**B**) Size distribution diagram and representative TEM image of Col M-exo and Mat M-exo. (**C**) Expression level of exosomal marker proteins, Alix, Tsg101, and MFG-E8, in various exosomes. (**D**) Comparison of exosome yield. Exosomes were extracted with an equal volume of cell-culture medium and milk. *n* = 5; *** *p* < 0.001 versus cell-culture medium.

With a minor modification of the ultracentrifugation extraction method of the previous study, [22] milk exosomes were obtained from commercialized mature milk and bovine colostrum (Figure 1A). First, milk was centrifuged at 5000× *g* for 30 min to separate the supernatant and milk fat globules (MFGs) from the solution. The supernatant was serially centrifuged at 12,000, 35,000, and 70,000× *g* for 1, 1, and 3 h, respectively, to remove milk fat, cell, cell debris, and casein. Then, the milk serum was sequentially filtered through 0.8, 0.45, and 0.2 μm pore size filters to eliminate bacteria and whey proteins. The filtered solution was ultracentrifuged at 100,000× *g* for 1 h to gain a milk exosome pellet. All processes of centrifugation were performed at 4 °C. The particle size and morphology of commercialized mature milk-derived exosomes (Mat M-exo) and colostrum-derived exosomes (Col M-exo) were verified by dynamic light scattering (DLS) and transmission electron microscopy (TEM), respectively (Figure 1B). The average diameter size of Mat M-exo and Col M-exo was 62.8 and 48.7 nm, respectively, and both exosomes presented a spherical morphology. The Western blotting analysis demonstrated that exosome marker proteins, Alix (exosome biogenesis proteins), Tsg101 (exosome biogenesis proteins), and MFG-E8 (milk exosome marker), were present in Col M-exos. Tsg101 and MFG-E8 were detected in Mat M-exo while Alix was scarcely detectable (Figure 1C). In bovine milk-derived exosomes, Alix is known to be present in higher abundance in the Col M-exo compared to the Mat M-exo [29]. In general, the yield of exosomes derived from animal cells including stem cells is known to be very low, which mainly limits their use [30]. Compared to cell-derived exosomes, all milk exosomes showed a significantly higher yield; ca. 20- and 40-fold increases in Mat M-exo and Col M-exo, respectively (Figure 1D).

In particular, the exosome yield from colostrum was 2.3 times higher than that from mature milk. The trait associated with super-high yield also confers competitive advantage in milk exosomes.

### 3.2. Absorption Rate and Cytotoxicity of Col M-exo into Skin Cells

**Figure 2:**
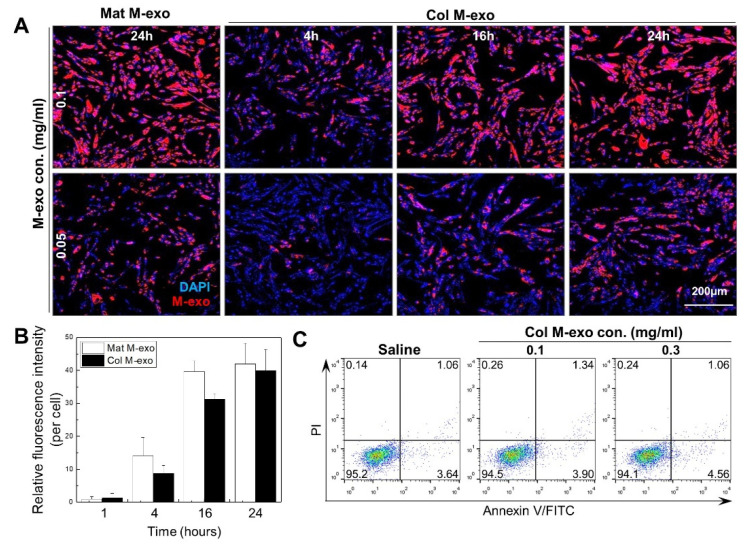
Cellular absorption tendency and cytotoxicity of milk exosomes. (**A**) Confocal microscopy image of HDFs after 4, 16, or 24 h incubation with 0.05 or 0.1 mg/mL of Cy5.5-labeled milk exosomes. Images of Cy5.5-labeled milk exosome (red) and DAPI (blue) were merged utilizing Leica Application Suite X software. Scale bar: 200 µm. (**B**) Relative fluorescence intensity per Cy5.5-labeled milk exosome-internalized HDFs. (**C**) FACS histogram displaying cytotoxicity of HDFs treated with 0.1 and 0.3 mg/mL of Col M-exo.

Prior to exploring the skincare effects of milk exosomes, we confirmed the tendency of exosomes labeled with Cy5.5-NHS to be absorbed inside human skin fibroblasts (HDFs) (Figure 2A). Intracellular absorption rate gradually increased according to the concentration and exposure time of Col M-exo and a similar pattern was also observed in the Mat M-exo-treated cells. As shown with the relative fluorescence intensity of Mat M-exo and Col M-exo (Figure 2B), their intracellular absorption enhancement reached a saturation at 24 h. Flow cytometry analysis with propidium iodide (PI)/Annexin V-FITC double staining was performed to investigate the cellular toxicity of milk exosomes (Figure 2C). The percentages of surviving (lower left), early-apoptotic (lower right), late-apoptotic (upper right), and necrotic (upper left) cells treated with different concentrations of Col M-exo were similar to that of the saline-treated control, indicating that milk exosomes cause no notable cytotoxicity on HDFs even at a high dose of 0.3 mg/mL. Based on these data, milk exosomes were used at concentrations of 0.1 mg/mL or less for further in vitro experiments.

Recently, exosomes derived from various cells have been reported to be involved in protein production, proliferation, and melanogenesis of skin cells [11,31,32]. Herein, we investigated the effects of milk exosomes on the prevention of UV-induced damage in representative skin cells such as keratinocytes, melanocytes, and fibroblasts. Cultured animal cell-derived exosomes, which were purified from three different types of skin cells, were used as control exosomes in each experiment.

### 3.3. Antioxidant Effect of Col M-exo on Keratinocytes

**Figure 3:**
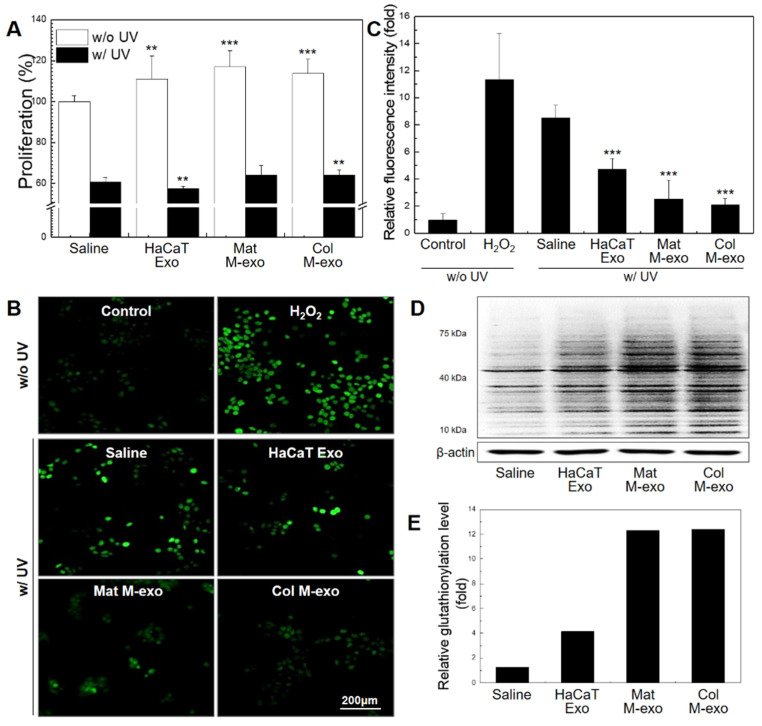
Antioxidant effect of milk exosomes on oxidative damage in HaCaTs. (**A**) Evaluation of cell proliferation rate of milk exosomes on HaCaTs was assessed by CCK-8 analysis. *n* = 5; ** *p* < 0.01, and *** *p* < 0.001 compared saline. (**B**) HaCaTs were pretreated with exosomes at a concentration of 0.05 mg/mL for 12 h. After UV irradiation, intracellular ROS levels were measured by DCF-DA assay. Scale bar: 200 µm. (**C**) Relative fluorescence intensity of intracellular DCF-DA. *n* = 3; *** *p* < 0.001 versus saline. (**D**) Western blot analysis of s-glutathionylated proteins. (**E**) Relative glutathionylation level using β-actin for normalization.

At low intracellular concentrations, ROS acts as an essential signal for regulation of normal physiological functions such as cell cycle, growth, and development [33,34]. Excess intracellular levels of ROS, however, are considered to damage all macromolecules, leading to activation of cell death processes. We used UV-C wavelength, the most harmful radiation, for UV cell damage experiment to more specifically classify UV-induced skin damage. To confirm the effect of milk exosomes on cellular ROS level in keratinocytes, HaCaTs were treated with exosomes from different sources: HaCaT Exo, Mat M-exo, and Col M-exo. Ahead of proceeding with ROS-related experiments, we assessed the relationship of exosomes with HaCaTs proliferation (Figure 3A). After treatment with different exosomes at a concentration of 0.1 mg/mL for 8 h, HaCaTs were further irradiated with UV-C for the with-UV group. Without UV irradiation, HaCaTs showed significantly increased proliferation levels by the treatment of HaCaT Exo (111.1 ± 11.3%), Mat M-exo (117.1 ± 7.8%), and Col M-exo (113.7 ± 7.1%). In general, exosomes that carry various bioactive cargos including proteins and RNA molecules are known to promote their own production and also stimulate cell proliferation [35,36]. After exposure to UV radiation, however, the treatment of exosomes exhibited no or only marginal effects on cell proliferation. Then, DCF-DA assay was used to confirm the relationship between the intracellular ROS level and milk exosomes (Figure 3B,C). HaCaTs were treated with each type of exosomes at a concentration of 0.05 mg/mL for 12 h and then the cells were irradiated with UV-C. After UV exposure, transiently increased intracellular levels of ROS were significantly decreased in Mat M-exo- or Col M-exo-treated HaCaTs. Although HaCaT Exo treatment somewhat improved antioxidant activity in UV-irradiated HaCaTs, both milk exosomes possessed a much stronger antioxidant activity (Figure 3C). Intracellular ROS is reduced by glutathione (GSH), resulting in glutathione oxidizing and glutathionylated protein [37]. Thus, we investigated the expression level of glutathionylated protein in HaCaTs treated with exosomes (Figure 3D,E). The expression level of glutathionylated protein in Mat M-exo- and Col M-exo-treated groups was significantly increased compared to saline and HaCaT Exo treatment group. Overall, milk exosomes appear to play a role in increasing the antioxidant capacity of HaCaTs by reducing intracellular ROS of HaCaTs through the pathway of glutathione oxidation. Given that macrophages are the major sources of ROS [38], we further examined the effect of Col M-exo on the oxidative stress in RAW264.7 cells. Similar results were found in the Col M-exo-treated macrophages, confirming the antioxidant effect of milk exosomes (Figure S1A).

It has been reported that the proliferation of keratinocytes is increased by exosomes obtained from induced pluripotent stem cell (iPSC)-derived mesenchymal stem cells (iMSCs) [11]. Moreover, human umbilical cord MSC-derived exosomes increase the expression of type 1 collagen in the wound and enhance proliferation and migration of skin cells through the Wnt/β-catenin pathway in vivo [16]. These MSC-derived exosomes have shown excellent therapeutic results, but have some major obstacles such as lack of exosome sources and low exosome yield. Interestingly, the exosomes derived from keratinocyte itself also increased proliferation in the normal state without UV irradiation but showed a similar or rather low proliferation rate as the control for exposure to UV radiation. On the other hand, milk exosomes showed some improvement in cell proliferation even under UV exposure. In particular, global intracellular protein glutathionylation dramatically increased in both groups treated with milk exosomes. These results suggest that milk exosomes increase resistance to oxidative stress by reducing ROS caused by UV-C through the GSH pathway, thereby maintaining the potency to induce proliferation of damaged cells.

### 3.4. Inhibitory Effect of Col M-exo on Melanogenesis in Melanocytes

**Figure 4:**
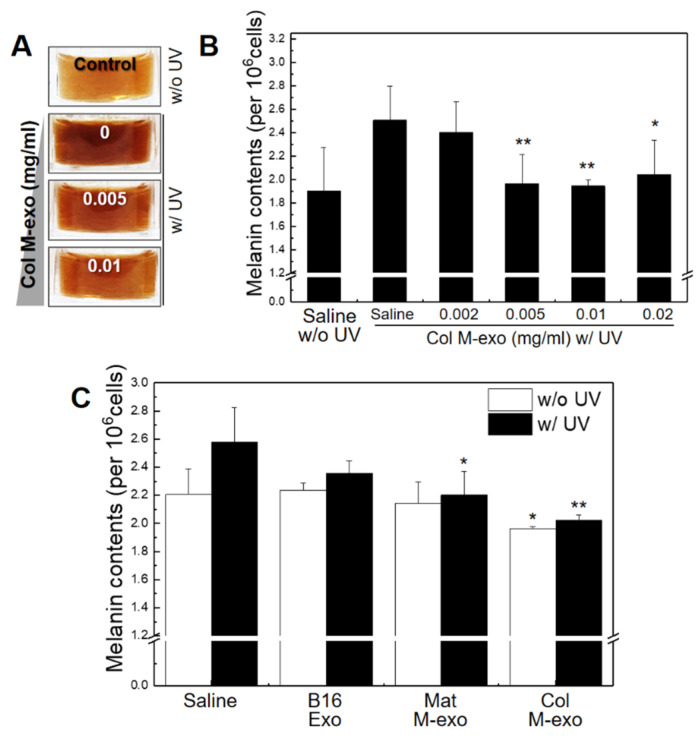
Inhibitory effect of Col M-exo on melanogenesis in B16F10s. (**A**) Image of extracting intracellular melanin from cells irradiated with UV after pre-treating cells with Col M-exo. (**B**) Melanin contents of cells irradiated with ultraviolet rays after pre-treating with Col M-exo at a concentration of 0.002, 0.005, 0.01, and 0.02 mg/mL, *n* = 3; * *p* < 0.05, ** *p* < 0.01, versus saline with UV. (**C**) Melanin contents of cells irradiated with UV after pre-treating with each type of exosome at concentrations of 0.01 mg/mL, *n* = 3; * *p* < 0.05, ** *p* < 0.01, versus saline.

Melanin is an important factor in determining human skin color and is produced from melanocytes by stimulation with UV rays. It plays an important role in protecting the skin against UV light damage. However, excessive melanin production can cause various dermatological disorders such as pigmentation and ephelides [39,40]. We used B16F10 cells instead of melanocytes as a model for the proliferation and melanin assay experiments. Thus, we performed a melanin assay to study whether milk exosomes can reduce melanin contents (measured as optical density (OD) at 490 nm) of B16F10s after irradiation with UV-C light. First, B16F10s were treated with Col M-exo at various concentrations for 24 h, and then the cells in the with-UV groups were irradiated with UV-C for 10 s (Figure 4A,B). In saline-treated B16F10s, UV exposure increased the melanin concentration (2.51 ± 0.29) compared to the control group without UV irradiation (1.90 ± 0.37). As expected, melanin production gradually dropped with increasing Col M-exo concentration. We further investigated the effect of different types of exosomes on the melanin content of B16F10s (Figure 4C). The melanin content of B16F10s was significantly reduced after milk exosome treatment without UV stimulation. Milk exosomes could also inhibit UV-induced melanin production. Specifically, Col M-exo was more effective to reduce melanogenesis than Mat M-exo at a concentration of 0.01 mg/mL.

Several previous studies reported that ingredients derived from milk are involved in the melanogenesis of melanocytes [41,42]. In addition, it is known that miRNAs (miRs) are strongly involved in the regulation of melanogenesis in melanocytes [43]. Milk exosomes are known to be rich in miR-7, 10, 21, 26, and 143, etc. [44]. miR-21 targets SOX5 and inhibits melanogenesis in UV-C irradiated melanocytes [45,46]. Moreover, miR-143 inhibits melanin production by reducing the expression of TGF-β-activated kinase 1 (TAK1) [47].

Considering these studies, it can be inferred that various exosomal cargo, including miRs and proteins, are attributed to attenuating melanogenesis.

### 3.5. Skin Elasticity Improvement Effect of Col M-exo on Fibroblasts

**Figure 5:**
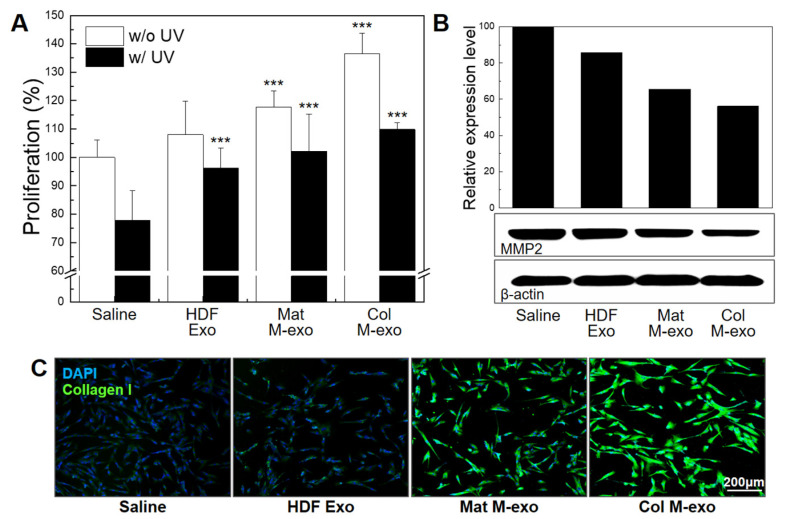
Skin elasticity improvement effect of milk exosomes on HDFs. (**A**) Proliferation rate of milk exosome-treated HDFs was assessed by CCK-8 analysis. *n* = 5; *** *p* < 0.001 versus saline. (**B**) Relative expression level of MMP2 proteins. (**C**) Representative immunostaining images of type 1 collagen (green) expressed in HDFs pretreated with Col M-exo for 24 h. Scale bar: 200 µm.

Fibroblasts are important factors for skin elasticity because they maintain the balance of the dermal microenvironment by synthesizing extracellular matrix (ECM) and collagen. Therefore, we used HDFs to investigate the relationship between milk exosomes and fibroblast growth (Figure 5A). HDFs were treated with each type of exosome for 8 h at a concentration of 0.1 mg/mL. The cells were further exposed to irradiated UV-C for UV stimulation. After 24 h incubation without UV irradiation, the proliferation rate of Mat M-exo- and Col M-exo-treated cells increased significantly by 117.6 ± 5.70% and 136.4 ± 7.26%, respectively. Under UV irradiation, milk exosomes also exhibited a strong cell proliferation-stimulating activity: the proliferation rates of Mat M-exo and Col M-exo treatment groups were 102.2 ± 13.1% and 109.9 ± 2.34%, respectively. In particular, exosomes extracted from healthy HDFs increased the proliferation rate by 96.2 ± 7.12% compared to saline (77.8 ± 10.5%), but there was no statistically significant increase in proliferation under normal conditions without UV stress. As mentioned above, exosomes are capable to support derived cell self-proliferation. Next, we analyzed the correlation between milk exosomes and matrix metalloproteinase-2 (MMP2) protein expression because MMP, an interstitial collagenase, degrades collagen and ECM, which impart tensile strength and elasticity to healed skin (Figure 5B). HDFs were treated with Col M-exo at a concentration of 0.1 mg/mL for 24 h, and then MMP2 protein expression was confirmed by Western blotting analysis. The expression levels of MMP2 in the cells treated with HDF Exo, Mat M-exo, and Col M-exo were decreased by 85.7, 65.6, and 56.1%, respectively. Subsequently, the protein expression level of type 1 collagen, one of major components of the dermal fibrils, was measured before and after exosome treatment (Figure 5C). Type 1 collagen was stained (Green) via immunocytochemistry and identified using a fluorescence microscope. The expression level of type 1 collagen was significantly increased with milk exosomes, especially Col M-exo in UV radiation-exposed fibroblasts. According to the previous studies, bovine milk induces type 1 collagen synthesis through the STAT6 pathway in human fibroblast [48] and donkey and human milk activate growth-regulatory kinases, especially the p-ERK pathway, promoting cell cycle and proliferation of skin fibroblasts in vitro [27]. Additionally, we conducted a wound-scratch test to confirm the migration promoting effect of Col M-exo. In particular, the enhanced migration result was found in the Col M-exo-treated group (Figure S2A). Consistent with our previous study that Col M-exo was rich in collagen and elastin deposition proteins and cell proliferation and migration-promoting proteins compared to Mat M-exo [49], the fibroblast migration ability was improved in the Col M-exo-treated group.

Taken together, these findings suggest that Col M-exo can effectively promote ECM construction by improving survival of skin fibroblasts and collagen production under UV irradiation, offering strength and elasticity to the skin.

### 3.6. Advantages of Col M-exo on Structural and Functional Stability

**Figure 6:**
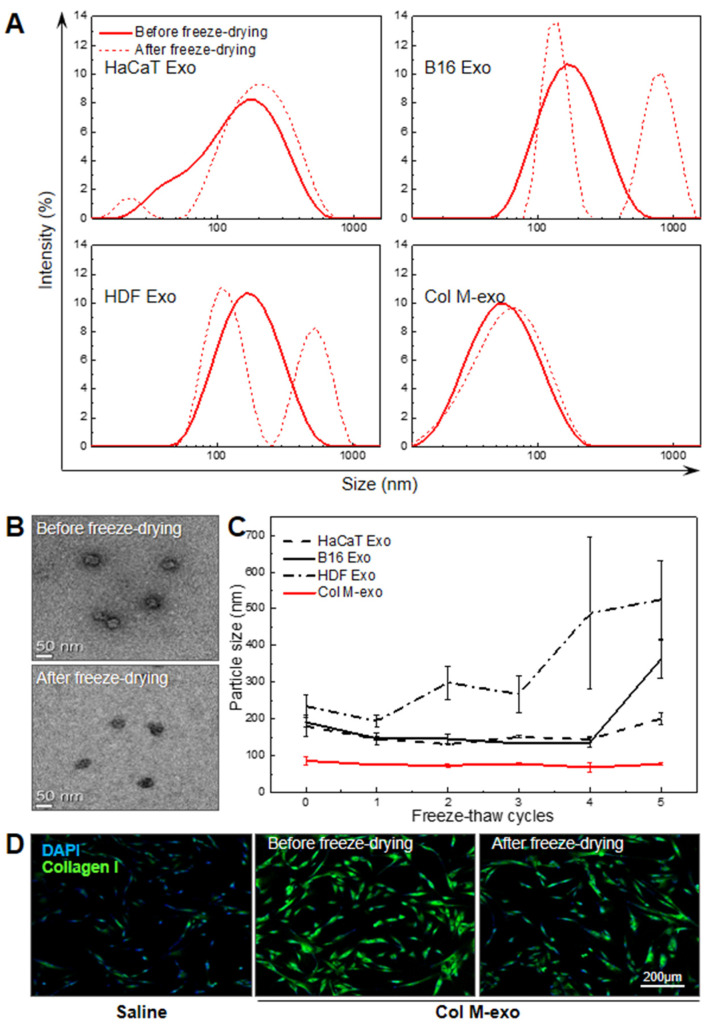
Structural and functional stability of Col M-exo. (**A**) Size distribution of exosomes extracted from cell-culture media and Col M-exo. Each type of exosome was compared before and after freeze-drying. (**B**) Representative TEM image of Col M-exo before and after freeze-drying. Scale bar: 50 nm. (**C**) Comparison of particle size distribution measurements of exosomes extracted from cell-culture media and Col M-exo according to freeze–thaw cycles. (**D**) Representative immunostaining images of type 1 collagen (green) in HDFs pretreated with Col M-exo before and after freeze-drying. Scale bar: 200 µm.

In addition to the super-high yield of milk exosomes, their thermal, mechanical, and functional stabilities are considered critical to be used as raw materials essential for derma therapeutics and cosmetics. According to previous studies, milk exosomes are structurally stable enough to pass through extreme pH environments and the GI tract [50]. To confirm the structural and functional stability of Col M-exo, we analyzed whether the original characteristics were maintained after the phase change of exosomes. Because the freeze-dried powder form is a suitable option for long-term storage of raw materials, we tried freeze-drying each type of exosome. Size of exosomes before and after freeze-drying was compared using DLS analysis (Figure 6A). The particle size of cell-derived exosomes showed a distribution of one peak before freeze-drying and changed to a distribution of two peaks after freeze-drying, whereas the size of Col M-exo remained almost unchanged with average diameters of ca. 56.8 nm after the lyophilization process. Similar to the DLS data, no considerable change occurred in the shape and size of Col M-exo with the TEM data before and after freeze-drying (Figure 6B). Additionally, we studied the effect of freeze–thaw cycles on the mechanical property of exosomes to verify their structural stability (Figure 6C). Unlike cell-derived exosomes, Col M-exo maintained a constant size up to five freeze–thaw cycles. These results clearly demonstrate that Col M-exo has higher structural stability than cell-derived exosomes in the rapidly-running reversible phase transition. Lastly, to analyze the functional stability of the lyophilized Col M-exo, the expression level of type 1 collagen (Green) in HDFs was confirmed using the immunocytochemistry method (Figure 6D). In the same manner as the natural form of milk exosomes, the lyophilized Col M-exo produced a similar stimulatory effect on type 1 collagen expression in fibroblasts. In addition, exosomes can pass through the stratum corneum barrier and be absorbed into the dermis, making them a suitable material for delivering active ingredients to skin cells.

Taken together, we were able to establish the advantages of the structural and functional stability of Col M-exo. These characteristics could be a beneficial advantage of transporting Col M-exo as essential raw materials applied in skin care products for the treatment or prevention of skin disorders.

## 4. Conclusions

In this study, we found that milk exosomes, especially Col M-exo, can be extracted dozens of times more than cell-derived exosomes from the same volume and have no detectable cytotoxicity even at high doses. Col M-exo have several beneficial effects on UV-induced aging and damage in resident skin cells. They can maintain their structural, mechanical, and functional properties after lyophilization and repeated freeze–thaw cycles, which is a potential benefit of using them as raw materials for advanced cell-free skin regeneration treatment. Milk is considered a biocompatible and safe substance that has been consumed by humans for thousands of years. Therefore, these outstanding physical properties, good cellular permeability, and anti-aging effects in skin cells make Col M-exo an attractive prospect for potential use as cosmetic active ingredients. In addition, engineered milk exosomes can be further utilized as an all-around-type carrier system for various types of drugs such as small molecule and nucleic acid drugs. Collectively, our findings indicate that bovine Col M-exo offer great potential as natural therapeutic agents to repair UV-irradiated skin aging and damage.

## Data Availability

All data available are reported in the article.

## Supplementary Materials

The following are available online at https://www.mdpi.com/article/10.3390/pharmaceutics14020307/s1, Figure S1: Antioxidant effect of Col M-exo on oxidative stress in RAW264.7 cells. (A) The intracellular ROS levels in RAW264.7 cells. RAW264.7 cells were pre-treated with Col M-exo at 0.1 mg/ mLfor 12 h and treated with 100 μM H_2_O_2_ for 30 min. After 6 h, the intracellular ROS levels were measured through DCF-DA assay, Figure S2: The promoting effect of Col M-exo on fibroblast migration. (A) Wound scratch migration assay in NIH-3T3 cells. NIH3T3 cells were treated with Mat M-exo or Col M-exo at a concentration of 0.1 mg/ mLfor 24 h. Thereafter, the cell migration area was measured using a microscope. *n* = 3; ** *p* < 0.01, *** *p* < 0.001 versus saline (24 h).
