# Supplementary material for: The Potential of Bovine Colostrum-Derived Exosomes to Repair Aged and Damaged Skin Cells
Source: Pharmaceutics. 2022 Jan 27;14(2):307. doi: 10.3390/pharmaceutics14020307 (PMC8877896; doi:10.3390/pharmaceutics14020307)
Supplement: Supplementary file 1 [file pharmaceutics-14-00307-s001.zip › pharmaceutics-1515137-supplementary.pdf]

pharmaceutics

MDPI

Supplementary Materials: The Potential of Bovine Colostrum-Derived Exosomes to Repair Aged and Damaged Skin Cells

Geonhee Han, Hyosuk Kim, Da Eun Kim, Yeonjoo Ahn, Joongsoo Kim, Ye Ji Jang, Kwangmeyung Kim, Yoosoo Yang and Sun Hwa Kim

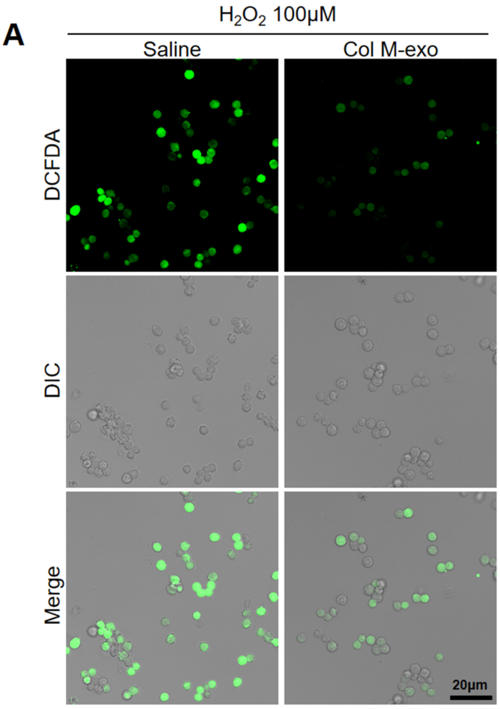

Figure S1. Antioxidant effect of Col M-exo on oxidative stress in RAW264.7 cells. A) The intracellular ROS levels in RAW264.7 cells. RAW264.7 cells were pre-treated with Col M-exo at 0.1 mg/ml for 12 h and treated with 100 μM H2O2 for 30 min. After 6 h, the intracellular ROS levels were measured through DCF-DA assay.

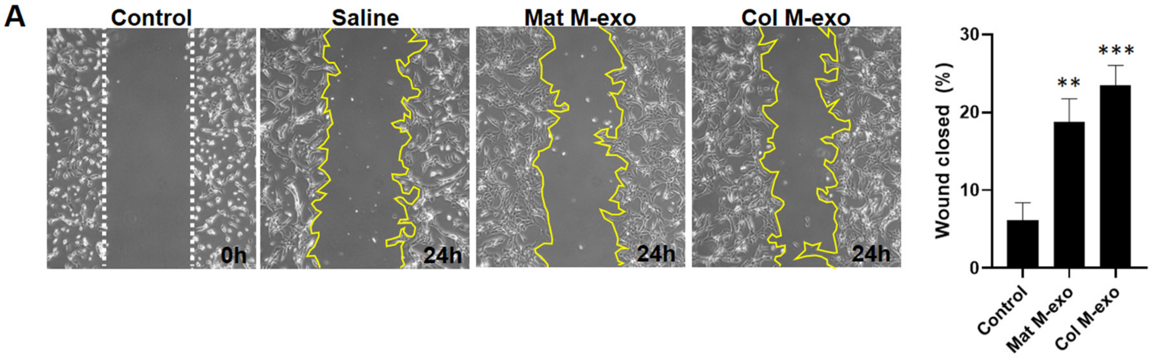

Figure S2. The promoting effect of Col M-exo on fibroblast migration. A) Wound scratch migration assay in NIH-3T3 cells. NIH3T3 cells were treated with Mat M-exo or Col M-exo at a concentration of 0.1 mg/ml for 24 h. Thereafter, the cell migration area was measured using a microscope. $n = 3$; $^{**}p < 0.01$, $^{***}p < 0.001$ versus saline (24 h).
